# Quantum Dots in Ocular Surface: A Systematic Review

**DOI:** 10.1002/wnan.70074

**Published:** 2026-08-18

**Authors:** Sidra Sarwat, Mark Willcox, Fiona Stapleton, Maitreyee Roy

**Affiliations:** ^1^ School of Optometry and Vision Science UNSW Kensington New South Wales Australia

**Keywords:** cornea, cytotoxicity, ocular surface, quantum dots, tear film

## Abstract

The current advancements in quantum dot‐based ocular surface applications identify critical gaps in existing research. A systematic search was conducted following PRISMA guidelines. Articles were retrieved from PubMed and Scopus using keywords including “quantum dots,” “semiconductor nanocrystals,” “ocular surface,” “tear film,” “cornea,” “conjunctiva,” “lacrimal glands,” and “meibomian glands” with Boolean conjunctions. A total of 232 studies were initially selected based on titles and abstracts. After removing duplicates and screening, 21 studies were selected for review. A variety of QDs, including carbon, graphene, silicon, cadmium and gold‐based, have been explored for bioimaging and therapeutic applications targeting the ocular surface. Carbon and silicon‐based QDs are the most biocompatible. Surface functionalization of these QDs facilitates targeted imaging of tear film layers, conjunctiva, and cornea with stable fluorescence and minimal toxicity. QDs can also be antimicrobial, anti‐inflammatory, and scavenge reactive oxygen species which might be used for the treatment of dry eye disease. Although cadmium‐based QDs exhibit superior fluorescence, their cytotoxicity limits their clinical application. The optical properties of QDs are influenced by physicochemical properties such as particle size, surface charge, and surface functionalization. QDs represent a promising class of nanoscale materials for diagnostic and therapeutic applications on the ocular surface. While biocompatible variants of QDs, such as carbon and silicon QDs, show potential for clinical use, there is limited information on the standardized synthesis protocols, long‐term cytotoxicity, systemic biodistribution, and clearance from ocular tissues.

This article is categorized under:

Nanotechnology Approaches to Biology > Nanoscale Systems in Biology

## Graphical abstract

Graphical abstract illustrating the types of QDs, their surface functionalization, diagnostic and therapeutic potential for ocular surface applications. This review highlights advances in QD‐based imaging of the tear film, conjunctiva, and cornea, emphasizing the role of biocompatible QDs in targeting inflammation, oxidative stress, and microbial activity, with a future focus on safety and standardization.

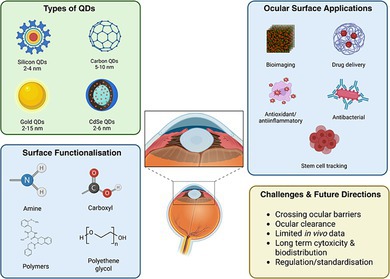

## 1 Introduction

Quantum dots (QDs) are semiconductor nanoparticles (2–20 nm in diameter) with size and composition‐dependent optical properties (Dabbousi et al. 1997). Their tunable fluorescence emission arises from quantum confinement effects, where smaller dots emit higher‐energy blue light (~450 nm) and larger ones emit lower‐energy red light (~700 nm) (Murphy 2002). This property, combined with superior photostability, enables prolonged imaging without photobleaching, a critical advantage for biomedical imaging (Arya et al. 2005). In addition, surface functionalization further enhances their use; QDs can be conjugated with antibodies, peptides, or DNA to target biological tissues (Matea et al. 2017; Michalet et al. 2005; Sahoo et al. 2008). These properties enable applications, tracking of cellular processes (Hoshino et al. 2004), real‐time bioimaging of tear film dynamics (Arya et al. 2005) and therapeutic systems (Matea et al. 2017). In ophthalmology, QDs have emerged as multifunctional tools with both diagnostic and therapeutic potential (Sarwat et al. 2019). Their nanoscale dimensions facilitate penetration through the corneal epithelium's tight junctions (De Hoon et al. 2023) while near‐infrared emissions (700–1600 nm) allow deep tissue imaging with minimal scattering (Dabbousi et al. 1997). For instance, hydrophobic silicon QDs (SiQDs) functionalized with scandium could selectively image the tear film's lipid layer (~40–100 nm) (Sarwat et al. 2025), whereas hydrophilic variants may label the aqueous phase, enabling simultaneous visualization of both layers (Sarwat et al. 2022). Real‐time slit‐lamp imaging has demonstrated the ability of QDs to track blink‐induced redistribution and drainage patterns, revealing previously unobservable interfacial dynamics (Khanal and Millar 2010). Beyond diagnosis, QDs have been developed as nanocarriers for ocular drug and gene delivery (Barnett et al. 2013; Olson and Mandava 2010; Olson et al. 2012; Takeda et al. 2009; Vranka et al. 2015) and integrated into antimicrobial formulations (El‐Gendy et al. 2022) for treating infectious keratitis (Jian et al. 2023) and conjunctivitis (Nur et al. 2020).

The ocular surface poses unique challenges: the precorneal tear film (3–5 μm thick) (King‐Smith et al. 2000) undergoes rapid turnover (~1–2 μL/min) (Khanal and Millar 2010), while the corneal epithelium's tight junctions (Maurice 1957) and conjunctival clearance mechanisms (Tam et al. 2011; Yücel et al. 2018) limit drug bioavailability. Conventional dyes, such as fluorescein, suffer from rapid photobleaching and broad emission spectra, which compromise resolution (Resch‐Genger et al. 2008). QDs may address these limitations through surface functionalization, such as coatings that enhance mucoadhesion, extending residence time (Abdelhameed et al. 2018; Zhang et al. 2007).

Recent advances prioritize the biocompatibility of QDs. Cadmium‐free QDs, such as SiQDs and carbon QDs (CQDs), reduce toxicity concerns (Sarwat et al. 2019). SiQDs doped with scandium or copper emit stable fluorescence and demonstrate excellent biocompatibility in human corneal epithelial cells (HCECs) at concentrations ≤ 16 μg/mL (Sarwat et al. 2025, 2022). Phase‐transfer methods using amphiphilic polymers render initially hydrophobic QDs water‐soluble, enabling clinical applications in biological systems (Sun et al. 2023). This systematic review critically evaluates the composition, synthesis, and functionalization of QDs for ocular surface applications. Preclinical data from in vitro, ex vivo, and in vivo models highlight their cytotoxicity. It also summarizes fluorescence properties, surface modifications, and biodistribution in terms of ocular barriers, the tear film, and the cornea. The review aims to highlight current advancements, identify knowledge gaps, and propose future directions for the clinical translation of QDs‐based nanotechnologies in ocular surface research.

## 2 Methods

**FIGURE 1:**
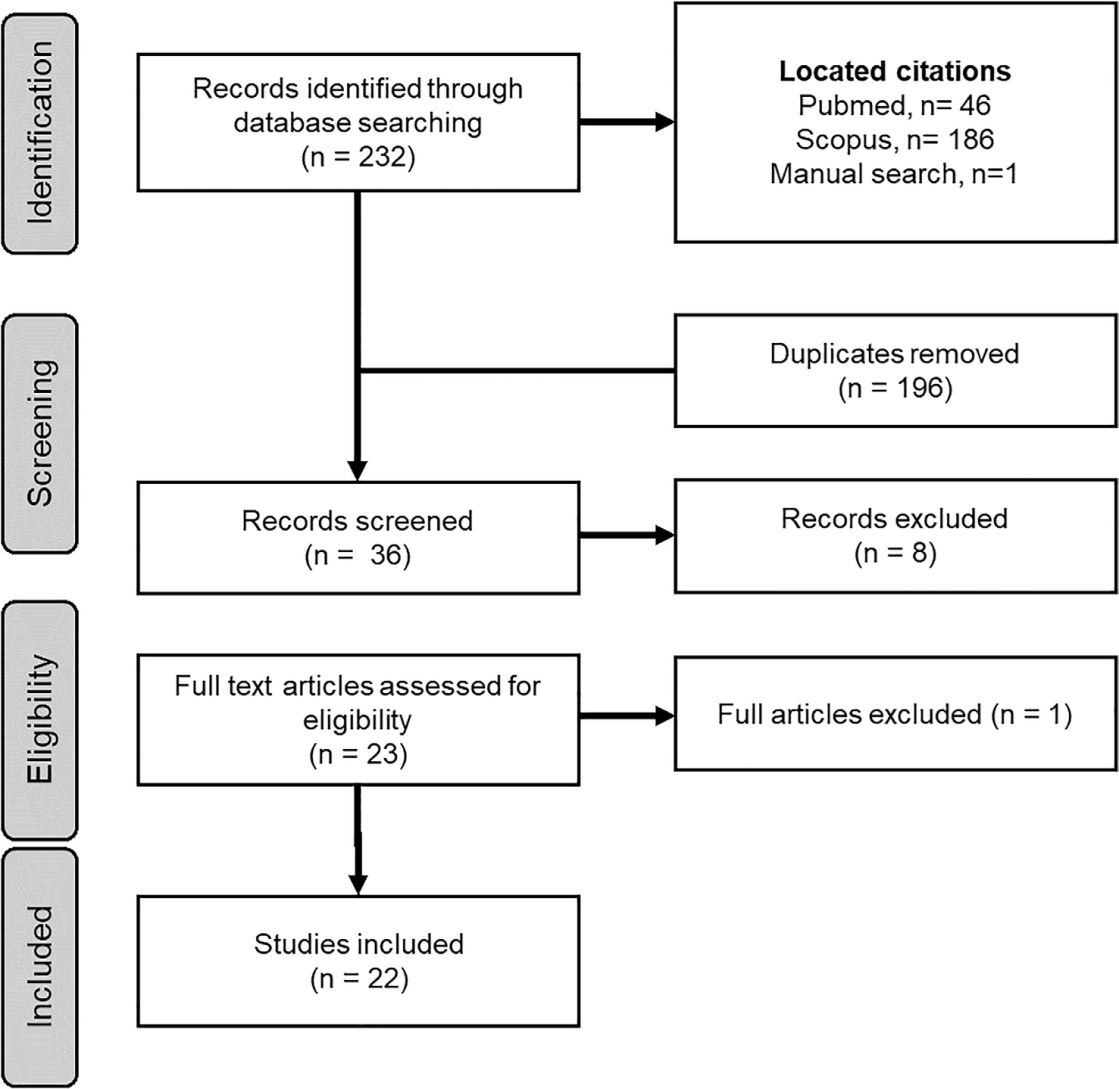
Flowchart of search, identification, and selection method of included studies.

This systematic review was conducted following the Preferred Reporting Items for Systematic Reviews and Meta‐Analyses (PRISMA 2020) guidelines. PubMed and Scopus were searched using keywords: QDs, semiconductor nanocrystals, ocular surface, tear film, dry eye disease, cornea, conjunctiva, lacrimal glands, meibomian glands, eyelids, up to May 2025. Boolean operators (AND/OR) were used to ensure comprehensive inclusion. Additionally, reference lists of eligible articles were manually screened to identify further relevant studies. A PRISMA 2020 flow diagram was generated to summarize the study selection process (Figure 1).

### 2.1 Inclusion Criteria

- Original, peer‐reviewed research articles published in English
- Studies investigating QDs (20 nm or less) applied to the ocular surface
- In vitro, ex vivo, in vivo (animal) experimental models or human studies

### 2.2 Exclusion Criteria

- Case studies, review articles, meta‐analyses or conference proceedings
- Studies involving secondary application of QDs to the ocular surface

An initial 232 records were identified (PubMed: 46; Scopus: 186). After removing duplicates and screening titles and abstracts, 36 full‐text articles were assessed. Of these, 22 studies met the inclusion criteria and were included in the final review. Each study was assessed for methodological quality based on the clarity of experimental design, reporting physicochemical characteristics of QDs, including cytotoxicity evaluation, and relevance to translational or in vivo application.

This review describes the current diagnostic and therapeutic applications of QDs on the ocular surface. Table 1 summarizes the characteristics of individual QDs used in the selected studies and their application in ocular surface research. Data extracted from each included study comprised the QD type, particle size, synthesis method, surface functionalization, target tissue, mode of administration, experimental model (e.g., HECs, rabbit eye, or mouse model), and outcomes related to cytotoxicity, biodistribution, imaging, and therapeutic applications. Additional references cited outside the selected studies were used for background, comparison, or discussion purposes and are separated from the systematic data extraction.

**TABLE 1:** Summary of studies using QDs for the ocular surface.

Study	QDs type	Synthesis method	Surface modification	Size (nm)	Fluorescence	Target tissue	Application	Model	Route	Cytotoxicity	Biodistribution
Sarwat et al. (2025)	Hydrophobic SiQDs	Solution phase	Hexane	2.65 ± 0.35	Green	Tear film lipid layer	Bioimaging	In vitro, ex vivo	Topical (modeled)	Low at 16 μg/mL	Not determined
Yu et al. (2025)	CQDs nano‐enzyme hydrogel	Microwave method	Polyethylene imine	4	Green	Tear film/DED model	Anti‐inflammatory, antioxidant therapy	Mouse	Topical (gel)	90% viability at 100 μg/mL	Retained on ocular surface for 96 h (3 days in vivo)
Zhang, Su, et al. (2024)	PQDs	Liquid‐phase exfoliation under nitrogen	Not determined	4.29 ± 1.4	Not determined	Corneal stromal cells	Anti‐inflammatory, anti‐fibrotic, regenerative therapy	In vitro and mice	Topical (hydrogel)	Nontoxic in vitro or in vivo over 28 days	Prolonged surface retention; effective ROS inhibition observed; no systemic spread indicated
Fu et al. (2024)	CQDs	Hydrothermal (amine passivation)	Amine‐rich surface	1.4 ± 0.5	465 nm	Salivary glands, ocular surface	Immune modulation	Mice	Intravenous	Low at 1 10 μg/mL	Accumulated in liver, lungs, kidneys, GI, and salivary glands
Wei et al. (2024)	CQDs nanozyme	Hydrothermal, embedded in gel	Enzyme‐embedded hydrogel	3 and 5	Green	Ocular surface	Antioxidant therapy for DED	Mice	Topical (solution and gel)	Biocompatible at 20 μg/mL for 7 days	Cleared within 20 min (solution), 130 min (gel)
Wu et al. (2024)	N‐GQDs	Hydrothermal	RGDS peptide (amine bond)	3.5 ± 1.2	Green (460 nm)	Corneal epithelium	Anti‐inflammatory, antioxidant therapy	Mouse	Topical	Low at 10 μg/mL in vivo/ex vivo	Gradual clearance; retained at cornea
Yang et al. (2024)	G‐CNQDs	Aqueous phase	Oxygen‐generating	5 and 8	Green	Cornea	Corneal crosslinking therapy	Rabbit	Topical	No toxicity at 800 μg/mL (in vitro)	Not determined
De Hoon et al. (2023)	CQDs	Hydrothermal	±surface charge	15	Blue‐Green	Cornea	Penetration and permeability	Ex vivo (porcine cornea)	Topical	IC50: 250 μg/mL (CQD‐S200), 500 μg/mL (others)	Positively charged QDs reached endothelium; Negatively charged QDs were limited to epithelium
Jian et al. (2023)	Arg‐CQDs	Polymeric Curcumin	Arginine and curcumin hybrid	29.3 ± 9.74/49.14 ± 19.8	Green	Ocular surface	Antibacterial hydrogel	Rats, rabbits	Topical	Low at 10 μg/mL	Retained in hydrogel; anterior chamber clear
Shilovskikh et al. (2023)	In based QDs	Commercial	Tobramycin conjugate	Not reported	Red (650 nm)	Anterior chamber	Penetration and antimicrobial	Rabbit	Topical	Not reported	Entered anterior segment
Wang et al. (2021)	CQDs in gel	Hydrothermal	Thermo‐sensitive in situ gel	2.02 ± 0.37, 90.12 ± 2.16	Blue	Ocular surface	Drug delivery, Imaging	In vivo, ex vivo	Topical (gel)	No irritation at 25100 μg/mL (Draize scale)	Retained in tears (30 min); cornea, lens, sclera, conjunctiva (10 min 2 h)
Nur et al. (2020)	CQDs (biosensor)	Not stated	Carboxyl	5 and 10	Red	Conjunctiva	Bioimaging	Rat	Topical	Not reported	Detected in eye (1 min), nose (20 min), limbs (60 min)
Sarwat et al. (2022)	Hydrophilic SiQDs	Wet chemical	Propyl amine	2.7 ± 0.4	Green	Tear film aqueous layer	Bioimaging	Ex vivo, in vitro	Topical	Low at 16 μg/mL	Not determined
El‐Gendy et al. (2022)	AuQDs	Laser Ablation in Liquid (Nd:YAG)	None specified (pure AuQDs)	7.8, 8.7, and 11.6	Not reported	Retinal epithelial cells, pathogens	Antimicrobial; combined laser and AuQD therapy	In vitro	Topical	Least cytotoxic at 7.8 nm: 86.8% cell survival at 20 μg/mL	Not assessed
Toda et al. (2019)	Not reported	—	Octaarginine (R8)	10 and 15	Red (680 nm)	Corneal endothelial cells	Cell tracking	Mice	Intracameral	< 8.0 nM (nontoxic)	Retained in corneal epithelium; later in lungs and liver
Qin et al. (2018)	CdSe/ZnS QDs	Not specified	Unmodified	6 and 13	525 and 655 nm	Corneal epithelial cells	Penetration under stress	In vitro (3D)	Topical	No toxicity observed	QDs penetrated epithelial layers
Jian et al. (2017)	CQDs (cationic)	Pyrolysis of spermidine	Super‐cationic	2 and 5	Green	Cornea	Antibacterial eye drops	In vitro and in vivo	Topical	No toxicity up to 100 μg/mL	Local accumulation in ocular tissue
Yang et al. (2015)	Not specified	Not reported	Not reported	Not reported	Not reported	Conjunctiva (engineered)	Cell differentiation	AECs	N/A	Non‐toxic	Trace in differentiated cells
Duncan et al. (2015)	CdSe/ZnS QDs	Commercial	COOH	3.28 ± 0.19, 14.98 ± 1.02	Green (525), Red (655)	Corneal epithelial sheets	Barrier function analysis	In vitro	Topical	Low	Size‐dependent penetration in corneal layers
Genicio et al. (2015)	CdSe/ZnS QDs	Tracking conjugate	Carboxylic/peptide	15 and 20	Bright Green (655)	Human limbal epithelial cells	Stem cell tracking	In vitro	Topical	No change in cell viability (10 μg/mL)	Cytoplasmic nuclear localization up to 2 weeks
Kuo et al. (2011)	CdSe/ZnS QDs	Commercial	PEG, amine, carboxyl	4.6	565 and 655 nm	Corneal stromal cells	Toxicity, distribution	In vitro & Mouse	IV and intrastromal	Viability reduced by 50% at 20 nM	Penetrated deep stroma; more with damaged epithelium
Khanal and Millar (2010)	InP/Ga/ZnS QDs	Commercial	Lipid (hydrophilic/hydrophobic)	20 and 25	Red	Tear film	Phase dynamics	Human	Topical	Not reported	Hydrophilic cleared via puncta; hydrophobic retained on surface

Abbreviations: Arg‐CQD: arginine‐functionalized carbon quantum dot, AuQD: gGold quantum dot, CdSe/ZnS QD: cadmium selenide/zinc sulfide quantum dot, CQD: carbon quantum dot, G‐CNQD: graphitic carbon nitride quantum dot, In‐based QD, Indium‐based quantum dot, InP/Ga/ZnS QD, Indium phosphide/gallium/zinc sulfide quantum dot, N‐GQD, nitrogen‐doped graphene quantum dot, PQD, phosphorus quantum dot, SiQD, silicon quantum dot.

## 3 Characteristics of QDS for Ocular Surface Applications

A total of 22 studies were included in this systematic review. The characteristics of the QDs and their specific applications on the ocular surface are summarized in Table 1.

### 3.1 Composition of QDs

Composition and structure of QDs are key determinants of their optical properties, biocompatibility, and functional performance in ocular applications.

#### 3.1.1 Cadmium‐Based QDs

Cadmium selenide (CdSe) cores with zinc sulfide (ZnS) shells remain widely studied for bioimaging and therapeutic delivery due to their tunable emission (450–660 nm) and high photoluminescence quantum yields (QYs) of 30%–50% (Duncan et al. 2015; Genicio et al. 2015; Kuo et al. 2011; Qin et al. 2018; Sarwat et al. 2019). Commercial CdSe/ZnS QDs achieve QYs > 70% with full‐width‐at‐half‐maximum (FWHM) values of 20–30 nm, critical for high‐contrast imaging (Grabolle et al. 2008). The ZnS shell passivates surface defects, reduces cadmium ion (Cd^2+^), leaching, and enhances photostability, enabling prolonged tracking of tear film dynamics (Dabbousi et al. 1997). Incorporating tellurium into CdSe cores extends emission to 670 nm (FWHM: 61 nm) with QYs of 45%, while zinc doping in CdZnS shells blue‐shifts absorption spectra for multiplexed imaging (Bai et al. 2022). For example, increasing zinc content in CdZnS shifts absorption spectra to shorter wavelengths, while incorporating selenium into CdSeS improves QYs by 69.5% through defect reduction (Chu et al. 2012). However, CdSe/ZnS QDs raise toxicity concerns due to the release of Cd^2+^, which induces oxidative stress and subsequent cell death (Kirchner et al. 2005). Similar QDs reduce corneal stromal cell viability by 50% at concentrations ranging from 5 to 20 nM over 24–48 h (Kuo et al. 2011).

#### 3.1.2 Cadmium‐Free Alternatives

Indium phosphide (InP) QDs with ZnSe/ZnS shells achieve unity QYs (100%) and narrow emission (FWHM: 32 nm) through hybrid shelling with zinc halides (e.g., ZnBr_2_) (Khanal and Millar 2010). Size progression from 3.9 nm (thin ZnSe shell) to 7.0 nm (thick ZnSe shell) enables emission tuning across 500–700 nm while maintaining biocompatibility in HCECs at < 16 μg/mL (Khanal and Millar 2010). Spherical gold QDs (AuQDs) (7.8–11.6 nm) synthesized via femtosecond laser ablation demonstrate antimicrobial efficacy against *Pseudomonas aeruginosa* and *Staphylococcus aureus* , with 86.8% HCEC viability at 20 μg/mL (El‐Gendy et al. 2022). Their surface plasmon resonance enhances photothermal activity, enabling combined laser‐QD therapies for infectious keratitis (El‐Gendy et al. 2022).

#### 3.1.3 Carbon and SiQDs

CQDs (De Hoon et al. 2023; Fu et al. 2024; Jian et al. 2017; Nur et al. 2020; Wang et al. 2021; Wei et al. 2024; Yu et al. 2025) and graphene QDs (GQDs) (Wu et al. 2024; Yang et al. 2024) are biocompatible alternatives for ocular applications. Arginine‐functionalized CQDs (29.3 ± 9.74 nm) show pH‐responsive fluorescence, enabling real‐time monitoring of tear film osmolarity changes in dry eye disease (Jian et al. 2023). The CQDs exhibit antioxidant properties (Wei et al. 2024; Wu et al. 2024), reducing oxidative stress (Yu et al. 2025) in ocular surface disorders while maintaining > 90% cell viability at 100 μg/mL concentrations (Yu et al. 2025). Nitrogen‐doped AuQDs (NAuQDs) (3.5 ± 1.2 nm) exhibit size‐dependent cellular uptake, demonstrating more effective penetration of corneal epithelial tight junctions than larger variants (Wu et al. 2024).

Scandium‐doped hydrophilic and hydrophobic SiQDs (2.65 ± 0.35) emit stable green fluorescence (*λ*_em_: 510 nm) for 20 min in artificial tears, enabling real‐time aqueous layer imaging (Sarwat et al. 2025, 2022). Both types maintain > 90% HCEC viability at concentrations > 16 μg/mL, though aggregation occurs above these concentrations due to surfactant destabilization (Sarwat et al. 2025, 2022). Comparative studies indicate non‐toxic profiles below this threshold in HCECs, with cytotoxicity linked to reactive oxygen species (ROS) generation rather than direct silicon toxicity (Kuo et al. 2011).

### 3.2 Optical Properties of QDs

The optical properties of QDs are determined by quantum confinement effects, where reduced particle size (1–20 nm) separates electronic energy levels, widening the bandgap and inducing a blue shift in fluorescence emission (Bardajee and Hooshyar 2013; Murphy 2002). This size‐dependent tuning enables precise control over emission wavelengths: smaller QDs (e.g., GQDs, CQDs, and SiQDs emit in the blue‐to‐green spectrum; 460–530 nm) (Fu et al. 2024; Sarwat et al. 2025, 2022; Wei et al. 2024), while larger core‐shell structures like CdTe/ZnS and InP/ZnSe/ZnS emit in the red‐to‐near‐infrared (NIR) range (605–700 nm) (Duncan et al. 2015; Khanal and Millar 2010), facilitating deep tissue imaging with reduced scattering and autofluorescence.

The photoluminescent properties of QDs are critically determined by their synthesis methodology (Brichkin and Razumov 2016). Bottom‐up approaches such as hydrothermal synthesis (Wang et al. 2021), microwave irradiation (Yu et al. 2025), and pyrolysis (Jian et al. 2017) offer precise control over QDs size and surface functionalities. For instance, hydrothermal synthesis produces nitrogen‐doped GQDs (NGQDs) with sustained green fluorescence (*λ*_em_: 510 nm, FWHM: 35 nm) and enhanced in vivo tissue retention (> 48 h postadministration) due to their zwitterionic surface charge (Wu et al. 2024). Pyrolytic methods yield CQDs that, when embedded in hyaluronidase‐sensitive hydrogels, maintain ocular surface fluorescence for 96 h in vivo via enzyme‐triggered payload release (Wei et al. 2024). Metallic QDs produced by laser ablation in liquid (LAL) showed potent red emission and antimicrobial performance with size‐dependent cytotoxicity (El‐Gendy et al. 2022). Solution‐phase synthesis allows control over hydrophobicity and emission lifetime, as demonstrated by hydrophobic SiQDs that maintained strong fluorescence in artificial tears for over 20 min (Sarwat et al. 2025, 2022).

### 3.3 Surface Functionalization of QDs

Surface functionalization modulates the optical properties of QDs (Abdelhameed et al. 2018). Unmodified QDs exhibit colloidal instability in aqueous media (aggregation index > 0.7 within 24 h) and nonspecific tissue interactions, which limit their diagnostic and therapeutic application (Qin et al. 2018). Covalent conjugation of functional groups (–COOH, –NH_2_, –OH) or polymers (e.g., PEG, pluronic F127) enhances aqueous dispersion (zeta potential: −25 to −40 mV) and reduces cytotoxicity (e.g., HCEC viability increases from 45% to > 90% at 50 μg/mL) (Duncan et al. 2015; Fu et al. 2024; Kuo et al. 2011; Nur et al. 2020; Yu et al. 2025). Ligand‐specific modifications, such as arginyl‐aspartic acid (RGDS) peptide, a sequence of amino acid that regulates cell adhesion and signaling, attachment to NGQDs, amplify fluorescence intensity (QY: 0.42 → 0.58) while improving corneal epithelial adhesion (residence time: 20 → 40 min) and antioxidant delivery (ROS reduction: 60% in dry eye models) (Wu et al. 2024). Similarly, enzyme‐responsive polymer coatings enabled QDs to provide controlled, stimulus‐sensitive emission within the tear film microenvironment (Wei et al. 2024).

## 4 Applications of QDs on the Ocular Surface

### 4.1 Bioimaging Applications of QDs

QDs enable high‐resolution, layer‐specific imaging of the tear film, conjunctiva and cornea due to their bright, size‐tunable fluorescence and surface modifiability (Nur et al. 2020; Sarwat et al. 2025, 2022; Toda et al. 2019). Commercially available InP/Ga/ZnS QDs have been employed to investigate tear film phase dynamics using both hydrophilic and hydrophobic SiQDs. Hydrophilic and hydrophobic SiQDs have been employed to visualize distinct layers of the tear film—specifically the aqueous and lipid layers—enabling high‐resolution analysis of tear film structure and dynamics (Sarwat et al. 2025, 2022). Carbon‐based QDs and CdSe/ZnS QDs have also been utilized for cellular and tissue‐level imaging, including imaging of the conjunctiva, corneal epithelial sheets, and stromal cells, as well as for tracking stem cells and epithelial differentiation (Duncan et al. 2015; Genicio et al. 2015; Nur et al. 2020). Furthermore, several studies have demonstrated the penetration and localization of QDs within ocular tissues, supporting their use in barrier function analysis and permeability studies under both normal and stressed conditions (De Hoon et al. 2023; Qin et al. 2018). These findings illustrate how QD formulations can be tuned for optimal imaging of the ocular surface structures.

### 4.2 Therapeutic Delivery Systems

The incorporation of QDs into drug delivery systems has enabled sustained release of therapeutic agents, including anti‐inflammatory and antioxidant therapies (Wu et al. 2024; Yu et al. 2025), immune modulation for dry eye disease (Fu et al. 2024), corneal crosslinking (Yang et al. 2015), antimicrobial delivery (Jian et al. 2017), and enhanced drug penetration (De Hoon et al. 2023; Qin et al. 2018). QDs facilitate cell tracking (Toda et al. 2019), stem cell differentiation (Yang et al. 2024), and barrier function analysis under physiological conditions (Duncan et al. 2015). Thermo‐responsive carbon dot hydrogels (90 nm composite structures) demonstrate pH‐dependent drug release kinetics critical for ocular surface applications (Wang et al. 2021). At physiological pH 7.4, the hydrogel maintains 89% drug encapsulation efficiency while acidic microenvironments (pH 5.5) in inflamed tissues trigger enhanced release within 2 h (Wang et al. 2021). Arginine‐functionalized CQDs (29.3 ± 9.74 nm) hybridized with curcumin exhibit dual antibiotic/anti‐inflammatory action, reducing *P. aeruginosa* biofilm viability by 4‐log units while suppressing TNF‐α production by 62% in rabbit models (Jian et al. 2017). Enzyme‐mimetic GQDs (3.5 ± 1.2 nm) engineered with RGDS peptide demonstrate targeted ROS scavenging capabilities (Wei et al. 2024). In murine dry eye models, these nanostructures reduce corneal ROS levels through catalase‐like and superoxide dismutase‐mimetic activities, while concurrently decreasing MMP‐9 expression by 58% via integrin‐mediated signaling modulation (Wei et al. 2024; Yu et al. 2025).

### 4.3 Antimicrobial Applications

AuQDs (7.8 nm) exhibit plasmon‐enhanced photothermal effects under 532 nm laser irradiation, generating localized temperature spikes of 52°C that eradicate methicillin‐resistant *S. aureus* biofilms within 5 min (El‐Gendy et al. 2022). Arginine–curcumin CQDs hydrogel exhibits potent antibacterial activity with minimal toxicity in ocular surface applications (Jian et al. 2017). The topical administration in rat and rabbit models demonstrates sustained retention within the hydrogel matrix and does not diffuse into the anterior chamber (Jian et al. 2017). At 10 μg/mL, the system exhibited low ocular toxicity while effectively inhibiting bacterial growth, confirming its suitability as a biocompatible antibacterial hydrogel (Jian et al. 2017). The nanoscale dimensions of these QDs optimize light absorption, achieving 3.2‐fold greater antimicrobial efficacy than 11.6 nm counterparts while maintaining 86.8% corneal epithelial cell viability at therapeutic concentrations (El‐Gendy et al. 2022). InP/ZnSe/ZnS QDs conjugated with tobramycin demonstrate size‐dependent anterior chamber penetration, with 6 nm particles achieving 89% higher aqueous humor antibiotic concentrations than free drug formulations (Shilovskikh et al. 2023). The quantum confinement effect enables real‐time tracking of drug distribution through near‐infrared fluorescence (*λ*_em_ = 650 nm), with signal intensity correlating linearly (*R* ^2^ = 0.94) with LC–MS quantified tobramycin levels (Shilovskikh et al. 2023).

### 4.4 Cellular Tracking and Regenerative Medicine

CdSe/ZnS QDs (15–20 nm) functionalized with limbal stem cell markers enable tracking of progenitor cell populations for up to 14 days in corneal regeneration models (Genicio et al. 2015). The large particle size prevents nuclear membrane penetration, ensuring cytoplasmic localisation that maintains 92% cell viability compared to 67% for nuclear‐targeted 4.6 nm QDs (Kuo et al. 2011). Graphitic carbon nitride QDs (5–8 nm) incorporated into artificial epithelial constructs enable real‐time monitoring of conjunctival goblet cell differentiation (Yang et al. 2024). Their oxygen‐generating property maintains normoxic conditions during tissue engineering, while size‐tuned fluorescence (*λ*_em_ = 510 nm) allows simultaneous tracking of MUC5AC expression and cell morphology changes (Yu et al. 2025). Hydrogel containing oxidized chitosan nanoparticles loaded with black phosphorus QDs inhibits the innate immune cascade fibrosis and promotes ocular surface reconstruction following chemical corneal injury (Zhang, Su, et al. 2024).

### 4.5 Clinical Translatability of QDs in Ocular Surface Applications

Despite promising preclinical findings, the clinical translatability of QDs for ocular surface applications remains limited. Most studies to date are based on in vitro and animal models, with no human clinical trials (Table 2). This research gap is related to the challenges of safety, reproducibility, and regulatory standardization.

**TABLE 2:** Clinical relevance of quantum dots in ocular surface diseases compared with conventional approaches.

Ocular surface disease	Current standard	Key limitations of the current approach	QDs‐based application	Advantage of QDs
Dry eye disease	Fluorescein staining, TBUT	Transient staining; no therapeutic effect; poor tear film dynamics assessment	Tear film imaging; antioxidant, and anti‐inflammatory therapy (CQDs, nanozyme hydrogels)	Prolonged retention, sustained drug release, real‐time monitoring
Tear film imaging	Fluorescein staining	Cannot differentiate lipid vs. aqueous layers; low spatial resolution	Layer‐specific imaging (SiQDs, InP/Ga/ZnS QDs)	High‐resolution, layer‐specific, dynamic imaging
Infectious keratitis	Microbial culture, topical antibiotics	Delayed diagnosis, non‐targeted therapy; no real‐time tracking	Antibacterial QDs (Arg‐CQDs, AuQDs)	Targeted antimicrobial delivery + imaging capability
Corneal injury/wound healing	Fluorescein staining, slit‐lamp exam	Only detects epithelial defects; no therapeutic role	Anti‐inflammatory, anti‐fibrotic QDs (PQDs, CQDs)	Combined imaging and regeneration
Corneal barrier/drug penetration	Fluorescein permeability tests	Limited mechanistic insight into drug transport	QD penetration studies (CQDs, CdSe/ZnS QDs)	Enables size/charge‐dependent mechanistic analysis
Ocular surface inflammation	Clinical grading, dyes	Subjective assessment; no targeted therapy	Anti‐inflammatory QDs (N‐GQDs)	Targeted, sustained therapeutic effect
Corneal crosslinking	UV‐riboflavin crosslinking	Oxygen dependency limits efficiency	Oxygen‐generating QDs (G‐CNQDs)	Enhanced, controlled crosslinking
Cell tracking/regeneration	Conventional fluorescent dyes	Photobleaching; short‐term tracking	QD‐based cell tracking	Long‐term stable fluorescence

Ocular surface diseases, including dry eye disease, infectious keratitis, and corneal epithelial injury, are characterized by tear film instability, inflammation, and epithelial disruption (Holland et al. 2013). Conventional treatments of these diseases are limited by poor bioavailability and rapid clearance of therapeutics, often requiring frequent dosing (Baranowski et al. 2014). QDs could offer unique advantages, including tunable optical properties, high photostability, and surface functionalization, enabling their use as both imaging probes and drug delivery systems (Sarwat et al. 2019). For example, amphiphilic or lipid‐modified QDs can enable real‐time, layer‐specific imaging of the tear film and improve therapeutic retention in dry eyes (Sarwat et al. 2025, 2022). In infectious keratitis, QDs may facilitate targeted antimicrobial delivery and allow real‐time monitoring of treatment response (Jian et al. 2017). Similarly, in corneal injury, QDs can support epithelial regeneration and enable continuous imaging of wound healing processes (Genicio et al. 2015).

Despite these advantages, biocompatibility remains a primary concern, particularly, for heavy metal‐based QDs, which may induce cytotoxicity, oxidative stress, and long‐term accumulation (Kirchner et al. 2005). Although CQDs and SiQDs demonstrate improved biocompatibility, long‐term ocular toxicity and biodistribution data remain limited (Chinnathambi et al. 2014; Zhang, Yang, et al. 2024). Additionally, nanoparticle design parameters, including size, surface charge, and functionalization, play a critical role in determining ocular tissue interactions, retention, and safety, underscoring the need for rational and standardized synthesis and design methods.

Future research should prioritize the development of biocompatible QDs, particularly carbon‐based platforms, alongside advanced surface functionalization such as PEGylation or zwitterionic coatings to minimize toxicity while maintaining imaging performance (Qi et al. 2012; Warnement et al. 2008). The design of ocular surface‐specific delivery systems with controlled release profiles, coupled with in vivo evaluation in disease‐relevant models, will be essential. The early‐phase clinical trials are required to validate safety and efficacy in humans, enabling the progression of QDs from experimental platforms to clinically viable solutions in ocular surface diagnostics and therapeutics.

## 5 Ocular Surface Barriers

### 5.1 The Anatomy of the Ocular Surface

**FIGURE 2:**
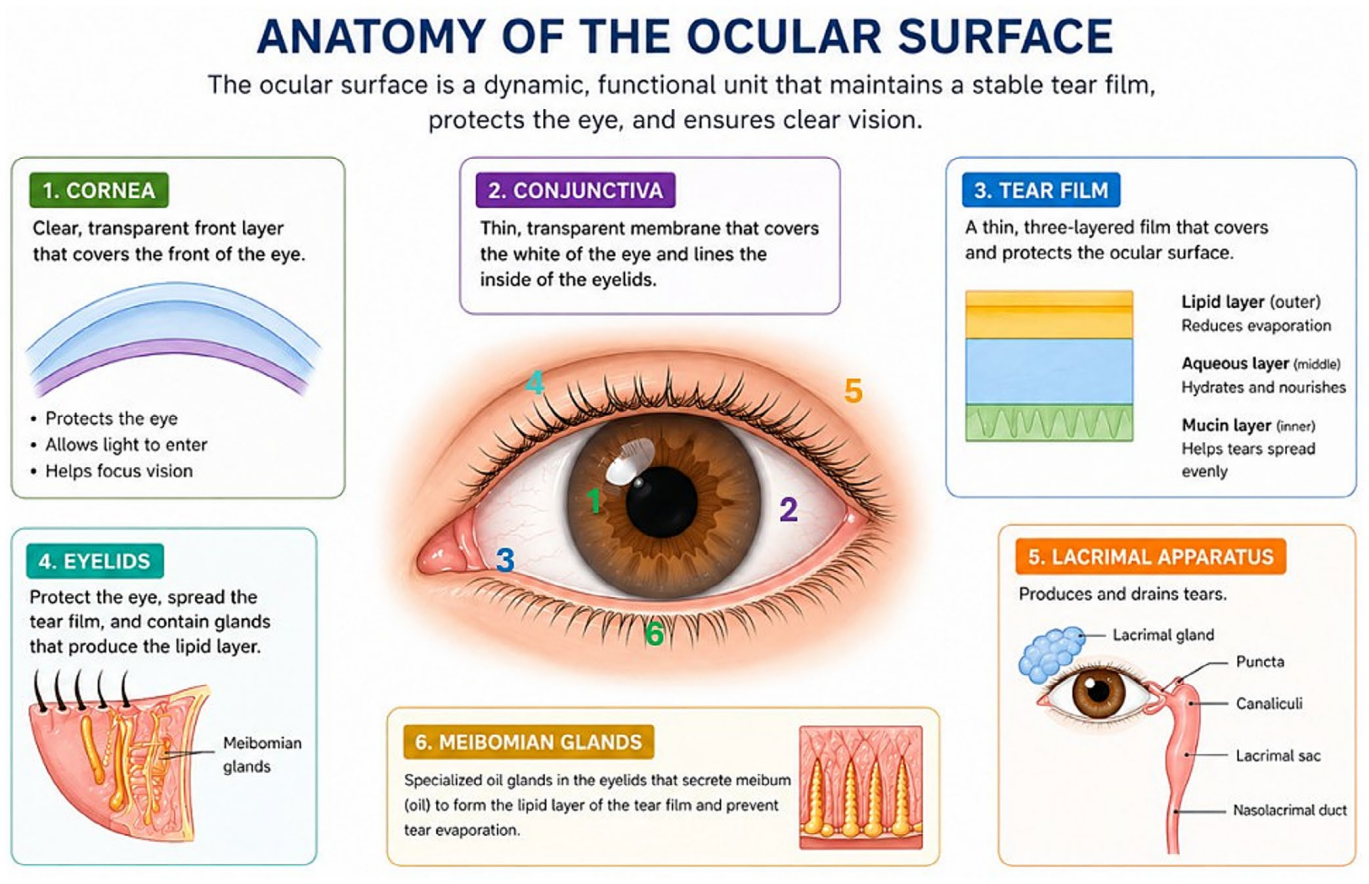
The anatomy of the ocular surface in the eye.

The ocular surface is a continuous mucosal and epithelial unit that includes the cornea, corneoscleral limbus, bulbar and palpebral conjunctiva, tear film, eyelid margins, meibomian and lacrimal glands, and the proximal lacrimal drainage system (Figure 2) (Gipson 2007). The transparent cornea forms the optical front of the eye and is a highly innervated protective surface, whereas the limbus is the transition zone between corneal and conjunctival epithelia and supports a stem cells population responsible for corneal renewal (Holland et al. 2013; Maurice 1957; Meek and Knupp 2015). The conjunctiva lines the posterior eyelids and merges onto the globe at the fornices; its goblet cells and vascular‐immune stroma contribute mucins, lubrication, and immune surveillance (Holland et al. 2013). Tears are produced by several cell types and glands, including the lacrimal gland (responsible for most of the aqueous component of tears), the meibomian glands in the eye's lids (which secrete the lipids of the tear film) and goblet cells in the conjunctiva (which secrete mucins into tears) (Willcox et al. 2017). Eyelid blinking spreads the tear film across the cornea and conjunctiva, while excess tears drain medially through the puncta, canaliculi, lacrimal sac, and nasolacrimal duct (Forst 1987). Together, these structures create a dynamic, tear‐washed interface rather than a static surface (Yanez‐Soto et al. 2014). This regional anatomy is important when interpreting ocular nanomaterial studies because topically applied particles could interact with tear turnover, blink mechanics, epithelial specialization, and drainage pathways before reaching cellular targets. These ocular structures and tear film characteristics could influence nanoparticle retention, clearance, and interaction with ocular tissues. Understanding this dynamic environment is essential for optimizing QDs design for effective imaging and drug delivery on the ocular surface.

### 5.2 Tear Film Barrier

The precorneal tear film is the outermost dynamic fluid in front of the eye (Dilly 1994; Willcox et al. 2017), influencing drug bioavailability through dilution, drainage, and clearance mechanisms (Benedetto et al. 1975). The tear film consists of an outer lipid layer (0.1 μm) secreted by meibomian glands, a middle aqueous layer (7 μm) produced by lacrimal glands, and an inner mucin layer (0.02–0.05 μm) derived from conjunctival goblet cells (Dilly 1994). The aqueous layer rapidly dilutes topically applied drugs, thereby reducing their effective concentration at the corneal surface (Tomlinson and Khanal 2005). Physiological tear turnover occurs at a rate of 1.2 μL per minute with a total tear volume of 7–10 μL, resulting in drug washout within 15–30 s of instillation (Garaszczuk et al. 2018). The nasolacrimal drainage further contributes to drug elimination, with approximately 80% of the instilled volume being cleared through the nasolacrimal duct within 5 min (Mishima et al. 1966). Additionally, the mucin layer creates a hydrophilic gel network that can impede drug diffusion while simultaneously providing a protective barrier that may interact with drug formulations through hydrogen bonding and electrostatic interactions, potentially altering drug release and corneal penetration (Hodges and Dartt 2013; Millar et al. 2006). To overcome these limitations, QDs are designed to interact with specific layer of the tear film (Khanal and Millar 2010; Sarwat et al. 2025, 2022). Similarly, hyaluronic acid‐conjugated CQDs (~15 nm) showed aqueous layer retention exceeding 130 min in dry eye models by binding to the corneal glycocalyx (Fu et al. 2024). Hydrophobic QDs, such as hexane‐functionalized SiQDs (~3 nm), have been shown to integrate into lipid layer of the tear film, exhibiting stable fluorescence for 30 min (Sarwat et al. 2025). Mucoadhesive QDs, such as polyethyleneimine‐coated CQDs (~4 nm), exhibit enhanced interaction with the mucin layer, resulting in up to 96‐h retention in enzyme‐sensitive hydrogels (Yu et al. 2025).

### 5.3 Corneal Barrier

The cornea is a complex, multilayered structure situated posterior to the tear film (Coco et al. 2024). Structurally, the main corneal barriers are epithelium, stroma, and endothelium, each exhibiting distinct biochemical and biophysical characteristics that collectively regulate drug permeation across the corneal barrier (Maurice 1957). The outermost layer, the corneal epithelium, consists of 5 to 7 layers of nonkeratinized stratified squamous epithelial cells interconnected via tight junctions, forming a dense lipophilic barrier approximately 50 μm thick (Meek and Knupp 2015). This epithelium effectively restricts the diffusion of hydrophilic molecules due to its lipid‐rich cellular architecture and the presence of tight intercellular junctions, thereby severely limiting paracellular transport pathways (Meek and Knupp 2015). Cationic CQDs (2–5 nm) facilitated tight junction imaging in the corneal epithelium due to their electrostatic interactions and subcellular‐scale resolution (Jian et al. 2017). In cases where the epithelial barrier is compromised or bypassed through drug conjugation strategies (e.g., tobramycin‐linked InP/ZnSe/ZnS QDs), enhanced anterior chamber penetration has been observed, with 6 nm QDs achieving 89% higher intraocular drug levels compared to free formulations (Shilovskikh et al. 2023). The underlying stroma, comprising 90% of corneal thickness (500 μm), consists of highly organized collagen fibrils embedded in a proteoglycan matrix with a high water content, creating a hydrophilic environment that impedes the diffusion of lipophilic drugs (Apostol and Carstocea 1994). Amphiphilic QDs with a diameter of ≤ 10 nm can potentially navigate these layers without compromising barrier integrity. For instance, 3.5 nm NGQDs have demonstrated up to 2.7‐fold greater stromal penetration than 15 nm cadmium‐based QDs, attributed to reduced steric hindrance (Wu et al. 2024). The innermost endothelium, a single layer of hexagonal cells with extensive tight junctions, regulates stromal hydration and presents an additional barrier to the movement of drugs into the anterior chamber (Apostol and Carstocea 1994; Meek and Knupp 2015). An optimal balance between lipophilic and hydrophilic properties is needed for effective trans‐corneal and tear film penetration (Coco et al. 2024). The tear film and cornea represent the primary barriers to topically administered drugs (Apostol and Carstocea 1994; King‐Smith 2016). To traverse the cornea, future QDs must be engineered with amphiphilic surface functionalization, balancing lipophilicity for epithelial passage and hydrophilicity for stromal diffusion.

## 6 Ocular Biodistribution of QDS

The size of QDs significantly influences their ocular retention and potential toxicity through interactions with anatomical and physiological barriers (Sarwat et al. 2019).

Particle size is a primary determinant of the fate of QDs following topical administration (Kim et al. 2009). Five nanometers CdSe/ZnS QDs undergo rapid nasolacrimal clearance, with 78% eliminated within 20 min due to tear turnover and lacrimal drainage (Khanal and Millar 2010). While this rapid clearance minimizes the risk of long‐term accumulation and potential tear film disruption, it limits their use for sustained imaging or drug delivery (Nur et al. 2020). QDs (5–15 nm), such as SiQDs (2.65 ± 0.35 nm), demonstrate improved corneal retention (45% at 60 min) by integrating into the lipid layer of the tear film without disrupting its 40–100 nm structure. Larger QDs (> 20 nm), however, are prone to mechanical entrapment in conjunctival folds, leading to localized accumulation and potential irritation (Wang et al. 2021). Thus, future QDs should utilize dual‐function surface coatings, mucoadhesive for binding to the tear film and hydrophobicity‐tuned for integration into lipid layers, while also maintaining colloidal stability.

Fifteen nanometer CQDs conjugated with hyaluronic acid exhibit aqueous layer retention times exceeding 130 min in dry eye models, facilitated by mucoadhesive interactions with the corneal glycocalyx (Fu et al. 2024). Cationic CQDs (2–5 nm) exploit size‐dependent quantum confinement effects to achieve subcellular resolution in corneal epithelial imaging (Jian et al. 2017). Their super‐cationic surface charge (+38.7 mV) drives electrostatic binding to anionic cell membranes, enabling real‐time (Wu et al. 2024) visualization of tight junction remodeling during epithelial stress responses (Jian et al. 2017). Smaller‐sized 3.5 nm NGQDs penetrate 2.7× deeper into stromal layers (Wu et al. 2024) than 15 nm cadmium‐based counterparts, attributed to reduced steric hindrance at nanoscale dimensions (Genicio et al. 2015). InP/ZnSe/ZnS QDs conjugated with tobramycin demonstrate size‐dependent anterior chamber penetration, with 6 nm particles achieving 89% higher aqueous humor antibiotic concentrations than free drug formulations (Shilovskikh et al. 2023). However, topical administration in rat and rabbit models demonstrates sustained retention within the hydrogel matrix and does not diffuse into the anterior chamber (Jian et al. 2017). InP/ZnSe/ZnS QDs conjugated with tobramycin demonstrate size‐dependent anterior chamber penetration, with 6 nm particles achieving 89% higher aqueous humor antibiotic concentrations than free drug formulations (Shilovskikh et al. 2023). The phosphorus QDs exhibited good biocompatibility in vitro and in vivo, with no significant cytotoxicity observed in corneal stromal cells over 6 days; live/dead staining and CCK‐8 assays showed minimal toxicity even at concentrations up to 100 μg/mL (Zhang, Su, et al. 2024). CdSe/ZnS QDs (15–20 nm) functionalized with limbal stem cell markers enable tracking of progenitor cell populations for up to 14 days in corneal regeneration models (Genicio et al. 2015). The large particle size prevents nuclear membrane penetration, ensuring cytoplasmic localization that maintains 92% cell viability compared to 67% for nuclear‐targeted 4.6 nm QDs (Kuo et al. 2011).

Surface modification is crucial for optimizing retention and minimizing cytotoxicity (Mehta et al. 2014). Polyethyleneimine‐coated CQDs (4 nm) use charge‐mediated mucoadhesion to 96‐h retention in enzyme‐sensitive hydrogels, though prolonged residence may alter tear film mucin‐lipid interactions, impacting ocular comfort (Yu et al. 2025). Hydrophobic modifications, such as hexane‐functionalized SiQDs, enhance lipid layer integration but require stabilization of the surfactant to prevent aggregation at concentrations exceeding 16 μg/mL (Sarwat et al. 2025). The hydrogel‐coupled phosphorus QDs were localized and retained on the ocular surface for up to 28 days without adverse effects or abnormal tissue responses (Zhang, Su, et al. 2024).

## 7 Systemic Biodistribution of QDS

QDs applied to the ocular surface exhibit distinct biodistribution and clearance patterns that depend on their size and composition. Smaller QDs (1.4–5 nm; e.g., carbon/silicon variants) demonstrate transient ocular retention (20 min–96 h) with limited systemic spread (Yu et al. 2025), while larger particles (> 15 nm; e.g., arginine‐CQDs at 29–49 nm) remain localized only in ocular tissues or hydrogels (Jian et al. 2023). Heavy‐metal QDs (CdSe/ZnS, 6–13 nm) penetrate epithelial barriers but show minimal systemic absorption (Qin et al. 2018), whereas indium‐based QDs migrate to the anterior chamber (Shilovskikh et al. 2023). Positively charged CQDs (15 nm) penetrate corneal layers, reaching the endothelium, while neutral/negative counterparts are restricted to superficial epithelia (De Hoon et al. 2023). CQDs accumulated in liver, lungs and kidneys after intravenous administration of ultrasmall (1.4 nm) QDs (Fu et al. 2024; Wei et al. 2024), whereas topical hydrogel‐embedded QDs (3–5 nm) prolong ocular surface retention (130 vs. 20 min for solutions) (Wei et al. 2024). Hydrophilic QDs (InP/Ga/ZnS, 20–25 nm) clear via tear drainage, while hydrophobic variants adhere to the ocular surface (Khanal and Millar 2010). AuQDs (7.8–11.6 nm) show size‐dependent cytotoxicity but no systemic migration (El‐Gendy et al. 2022). Surface modifications like RGDS peptides (3.5 nm GQDs) enhance corneal adhesion (Wu et al. 2024), while hydrogel matrices delay elimination to 96 h without systemic toxicity (Yu et al. 2025).

## 8 Cytotoxicity of QDS

Cadmium‐based QDs pose significant toxicity risks due to ion leaching (Kirchner et al. 2005). Thin zinc sulfide (ZnS) shells permit Cd^2+^ release within 72 h, reducing corneal stromal cell viability by 50% at 5–20 nM concentrations (Grabolle et al. 2008). Thicker shells (5 monolayers) reduce leaching to < 2%, preserving > 90% cell viability (Grabolle et al. 2008). Unpassivated CdSe cores enhance oxidative stress by generating hydroxyl radicals, thereby disrupting lipid peroxidation in the tear film (Wang et al. 2021).

Heavy metal‐free alternatives offer safe alternatives. Indium phosphide/zinc selenide/zinc sulfide (InP/ZnSe/ZnS) QDs achieve near‐unity QYs (94%–100%) with minimal ion leaching, showing no histopathological changes at 16 μg/mL. NGQDs exhibit 58% QY and 90% HCEC viability at 100 μg/mL (Wu et al. 2024), while AuQDs demonstrate 86.8% viability at 20 μg/mL alongside antimicrobial efficacy (El‐Gendy et al. 2022). Positively charged QDs enhance corneal epithelial adhesion (e.g., spermidine‐coated CQDs) but may induce oxidative stress via ROS generation while negatively Charged QDs limit corneal penetration due to electrostatic repulsion with the negatively charged epithelium but reduced cellular uptake and toxicity (De Hoon et al. 2023). Consequently, future ocular QDs should favor cadmium‐free core compositions, low‐leaching shells, and tailored surface charge profiles to balance corneal uptake with minimal oxidative stress or immunogenicity.

## 9 Clearance of QDS

The clearance of QDs from the ocular surface is a critical factor influencing their retention, bioavailability, and overall therapeutic performance. Following topical administration, QDs are exposed to a highly dynamic pre‐corneal environment, where tear turnover, blinking, and nasolacrimal drainage contribute to rapid dilution and elimination. These physiological processes present a major barrier to achieving effective ocular delivery and sustained interaction with the ocular surface tissues. A critical barrier to ocular drug delivery lies in the complex pre‐corneal pharmacokinetics of ocular drug delivery (Bu et al. 2007).

The ocular surface presents multiple dynamic physiological barriers, including tear film turnover (basal rate 10%–20% per minute) and the nasolacrimal drainage system, which together result in rapid dilution and elimination of topically administered agents (Garaszczuk et al. 2018). It has been reported that up to 60% of an instilled dose may be cleared within the first 2 min, significantly limiting drug bioavailability (Mooi et al. 2017). The clearance of nanomaterials is strongly influenced by their physicochemical properties, including particle size, surface charge, and surface functionalization (Meng et al. 2013). For instance, smaller nanoparticles (50 nm) may exhibit enhanced epithelial penetration but are also more susceptible to systemic clearance, whereas larger particles (> 100 nm) may demonstrate prolonged residence time on the ocular surface.

**FIGURE 3:**
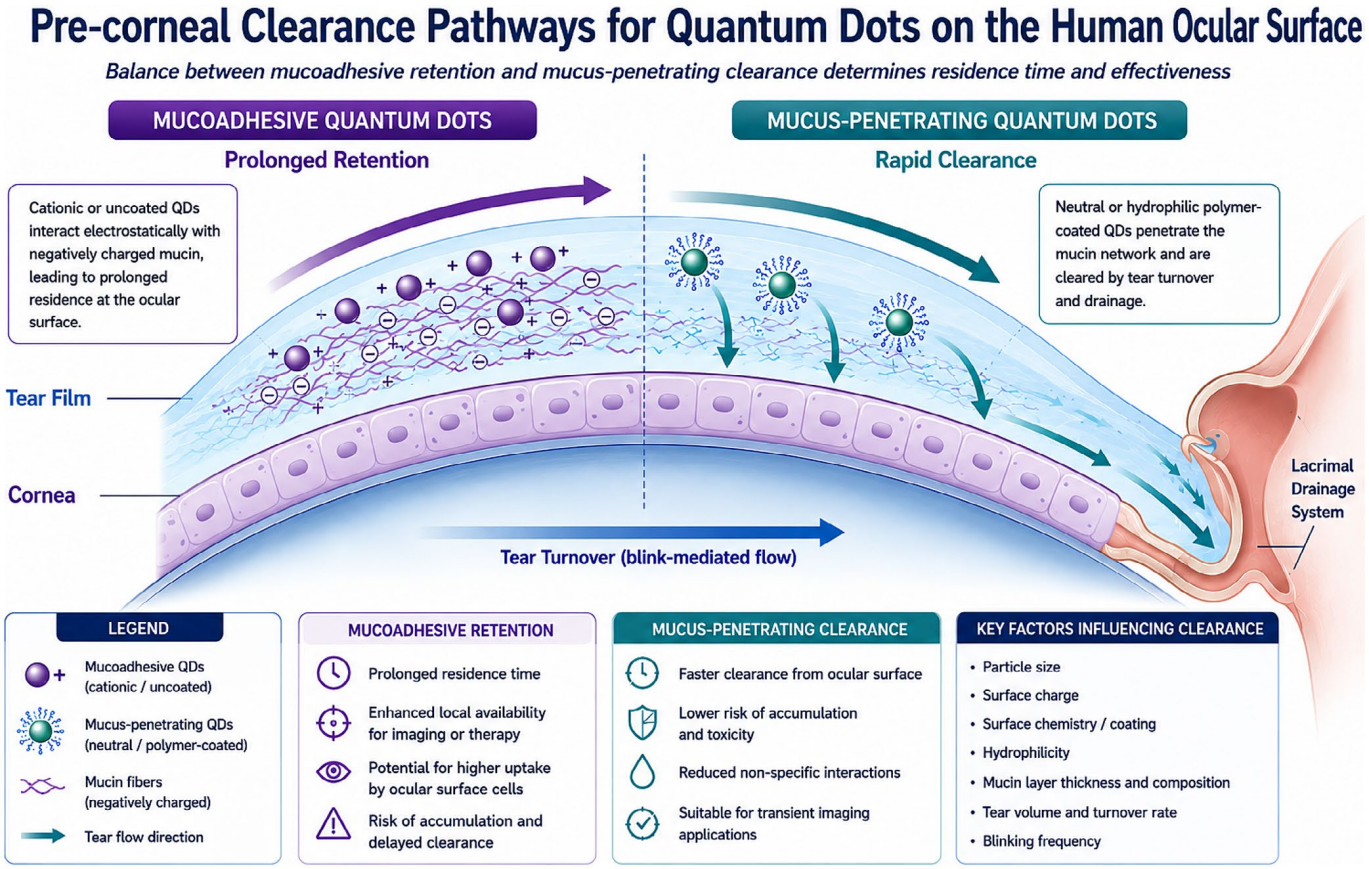
Clearance pathways for QDs on the human ocular surface.

To overcome these challenges, mucoadhesive systems, often employing cationic polymers such as chitosan, could enhance retention via electrostatic interactions with the negatively charged mucin layer, thereby prolonging ocular residence time (Figure 3). In contrast, mucus‐penetrating particles (MPPs), typically functionalized with neutral hydrophilic coatings such as polyethylene glycol (PEG), are designed to minimize interaction with mucins and facilitate diffusion through the mucus barrier to reach underlying epithelial tissues (Figure 3) (Qi et al. 2012). These advanced delivery systems are increasingly evaluated using multicompartment pharmacokinetic models, which simulate nanomaterial transport across ocular compartments (e.g., tear film and anterior chamber) to predict bioavailability and therapeutic efficacy.

## 10 Conclusions

**FIGURE 4:**
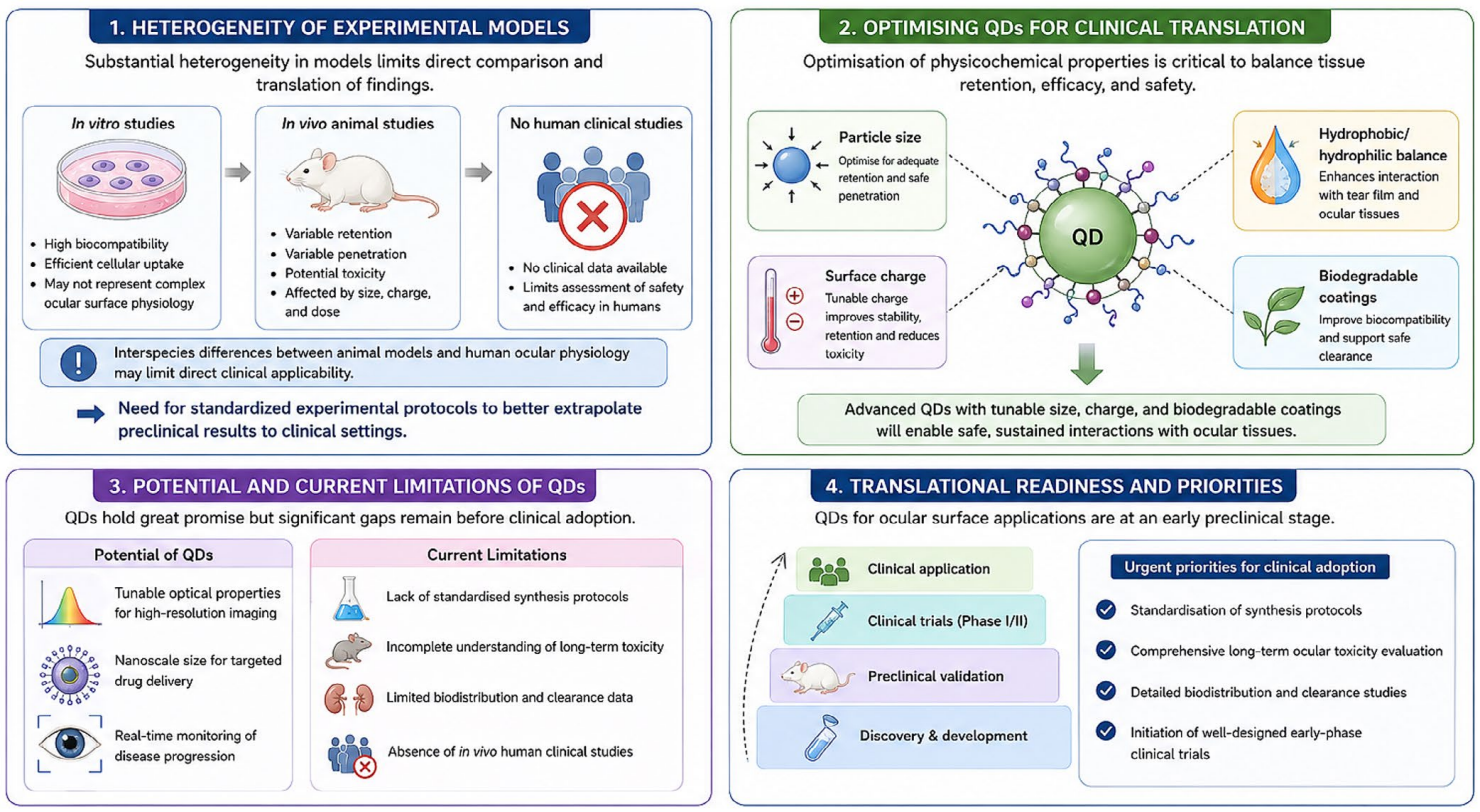
Summary of the major conclusions, current limitations, and translational considerations of QDs for ocular surface applications.

A key limitation across the included studies is the substantial heterogeneity in experimental models, which makes it difficult to compare and interpret findings directly. Most studies were conducted in vitro or in animal models, with no human clinical studies identified. While in vitro studies frequently report high biocompatibility and efficient cellular uptake of QDs, these findings may not fully represent the complex physiology of the ocular surface environment, including tear film dynamics, blinking, and immune responses (Figure 4). In contrast, in vivo studies demonstrate variable outcomes in terms of retention, penetration, and potential toxicity, influenced by physicochemical properties such as particle size, surface charge, and dosing. Furthermore, interspecies differences between animal models and human ocular physiology may limit the direct clinical applicability of these findings. These inconsistencies in experimental models highlight the need for standardized experimental protocols while extrapolating preclinical results to clinical settings.

The clinical translation of QDs for ocular surface imaging and therapy depends on optimizing particle size and surface chemistry to balance tissue retention and safety. Incorporating tunable surface charge, hydrophobic/hydrophilic balance, and biodegradable coatings will be key to developing advanced QDs for safe, sustained QDs ocular tissue interactions, thereby ensuring the safe integration of QDs into ophthalmic applications.

QDs, particularly biocompatible variants such as carbon‐ and silicon‐based QDs, represent a potential for advancing ocular surface diagnostics and therapeutics. Their tunable optical properties, nanoscale dimensions, and surface functionalization make them ideal for high‐resolution imaging, targeted drug delivery, and real‐time monitoring of disease progression. However, the clinical translation of QDs remains limited by the absence of standardized synthesis protocols, incomplete understanding of long‐term toxicity, biodistribution, and insufficient data on in vivo clearance mechanisms. Ocular‐surface QDs demonstrate strong potential for high‐resolution tear film imaging and targeted drug delivery; however, their clinical translation remains limited due to safety concerns, lack of standardization, and absence of clinical validation. At present, the translational readiness of QDs for ocular surface applications remains at an early preclinical stage, and urgent priorities for clinical adoption include standardization of synthesis protocols, comprehensive long‐term ocular toxicity evaluation, detailed biodistribution and clearance studies, and the initiation of well‐designed early‐phase clinical trials.

## Author Contributions

**Sidra Sarwat:** conceptualization (equal), data curation (equal), formal analysis (equal), methodology (equal), writing – original draft (equal), writing – review and editing (equal). **Mark Willcox:** project administration (equal), supervision (equal), visualization (equal), writing – review and editing (equal). **Fiona Stapleton:** project administration (equal), supervision (equal), visualization (equal), writing – review and editing (equal). **Maitreyee Roy:** conceptualization (equal), methodology (equal), project administration (equal), resources (lead), supervision (lead), visualization (equal), writing – review and editing (equal).

## Funding

The authors have nothing to report.

## Conflicts of Interest

The authors declare no conflicts of interest.

## Related WIREs Articles

Fundamentals, challenges, and nanomedicine‐based solutions for ocular diseases

## Data Availability

Data sharing is not applicable to this article as no new data were created or analyzed in this study.
